# Characterisation of LTR-Retrotransposons of *Stevia rebaudiana* and Their Use for the Analysis of Genetic Variability

**DOI:** 10.3390/ijms23116220

**Published:** 2022-06-01

**Authors:** Samuel Simoni, Clarissa Clemente, Gabriele Usai, Alberto Vangelisti, Lucia Natali, Silvia Tavarini, Luciana G. Angelini, Andrea Cavallini, Flavia Mascagni, Tommaso Giordani

**Affiliations:** Department of Agriculture, Food and Environment (DAFE), University of Pisa, Via del Borghetto 80, 56124 Pisa, Italy; samuel.simoni@phd.unipi.it (S.S.); clarissa.clemente@phd.unipi.it (C.C.); alberto.vangelisti@agr.unipi.it (A.V.); lucia.natali@unipi.it (L.N.); silvia.tavarini@unipi.it (S.T.); luciana.angelini@unipi.it (L.G.A.); andrea.cavallini@unipi.it (A.C.); flavia.mascagni@unipi.it (F.M.)

**Keywords:** *Angela* retrotransposon, Inter-Retrotransposon Amplified Polymorphism, LTR-retrotransposons, retrotransposon dynamics, *Stevia rebaudiana*

## Abstract

*Stevia rebaudiana* is one of the most important crops belonging to the Asteraceae family. Stevia is cultivated all over the world as it represents a valid natural alternative to artificial sweeteners thanks to its leaves, which produce steviol glycosides that have high sweetening power and reduced caloric value. In this work, the stevia genome sequence was used to isolate and characterise full-length long-terminal repeat retrotransposons (LTR-REs), which account for more than half of the genome. The *Gypsy* retrotransposons were twice as abundant as the *Copia* ones. A disproportionate abundance of elements belonging to the *Chromovirus/Tekay* lineage was observed among the *Gypsy* elements. Only the *SIRE* and *Angela* lineages represented significant portions of the genome among the *Copia* elements. The dynamics with which LTR-REs colonised the stevia genome were also estimated; all isolated full-length elements turned out to be relatively young, with a proliferation peak around 1–2 million years ago. However, a different analysis conducted by comparing sequences encoding retrotranscriptase showed the occurrence of an older period in which there was a lot of LTR-RE proliferation. Finally, a group of isolated full-length elements belonging to the lineage *Angela* was used to analyse the genetic variability in 25 accessions of *S. rebaudiana* using the Inter-Retrotransposon Amplified Polymorphism (IRAP) protocol. The obtained fingerprints highlighted a high degree of genetic variability and were used to study the genomic structures of the different accessions. It was hypothesised that there are four ancestral subpopulations at the root of the analysed accessions, which all turned out to be admixed. Overall, these data may be useful for genome sequence annotations and for evaluating genetic variability in this species, which may be useful in stevia breeding.

## 1. Introduction

Repeated sequences constitute a large part of the eukaryotic genome, especially in species with large genomes. They include tandem-arranged satellite sequences, ribosomal DNA, telomeric DNA sequences, and, mostly, transposable elements (TEs) [1]. TEs can autonomously replicate and move across different parts of the host genome [2]. They belong to two major classes, Class I or retrotransposons (REs) and Class II or DNA transposons, according to the mechanism by which they transpose. Class I elements transpose through a “copy and paste” mechanism, using an RNA molecule as an intermediate for replication; instead, Class II TEs move through a “cut and paste” mechanism [3].

The most ubiquitous and abundant TEs are notoriously REs, especially those with long-terminal repeats (LTRs). RE abundance is related to their “copy and paste” mechanism of transposition, by which the element is first transcribed into an RNA molecule, then retro-transcribed to DNA, and finally inserted at another locus in the genome, determining an increase in RE copy number [2]. REs range in size from a few hundred base pairs to over 10 kb [2], and in the case of complete autonomous elements, they include a coding portion containing two open reading frames (ORFs) to be used for the replication and integration of the element in the host genome [4]. The coding portion is flanked by the two LTRs. The two ORFs include *pol*, which encodes a polyprotein (with a protease, a reverse transcriptase, an RNaseH, and an integrase enzyme domain), and *gag*, which encodes a virus-like particle structural protein. Autonomous REs have all of these enzyme domains, which are necessary for transposition. In cases where one or more enzyme domains are missing, non-autonomous LTR-REs can use enzymes produced by autonomous REs to retrotranspose [2].

Although REs have long been considered not to provide adaptive advantages to the host species and have therefore been termed “selfish” DNA [5], it is now largely accepted that REs have considerably contributed to the evolution of the genome of eukaryotic species. In addition to polyploidy, LTR-RE transposition is a key mechanism that produces genome size variation [6]. REs are involved in genome restructuration favouring chromosome structural changes; they supply chromatin boundary signals for heterochromatin domains, playing a central role in structuring the nucleus [7]. Even more importantly, RE repeats have a primary role in modifying the host’s regulatory network and, consequently, gene expression [8]. The insertion or loss of an LTR-RE can change promoters’ and enhancers’ structures, possibly altering the regulatory patterns of coding regions and leading to phenotypic variations [9,10,11,12]. Finally, LTR-RE insertion or loss can alter the phenotype, changing the epigenetic setting of a genetic locus, with consequences for chromatin organisation and the expression of adjacent genes [7,13].

The sequence similarity of LTR-REs among species is often minimal and limited to coding regions [2]. Plant LTR-REs are subdivided into two major superfamilies, *Gypsy* and *Copia*, according to sequence and structural similarities. The most striking difference between the two superfamilies refers to the linear order of the enzyme domains along the Pol protein. In angiosperms, many *Gypsy* and *Copia* lineages have been identified [14,15,16,17,18,19]. The main *Gypsy* lineages are *Chromovirus*, a lineage of REs carrying a chromodomain at the 5′ end of the coding portion, which are especially abundant in centromeres [15,20], and non-*Chromovirus*, subdivided into *Athila* and *Tat* and represented by large elements with a further open reading frame located upstream of the *gag* gene [19]. *Copia* LTR-REs can also belong to many different lineages, the most diffused being *Ale*, *Ivana*, *Angela*, *Bianca*, *TAR*, and *SIRE* [14,19,21].

In this work, we aimed to characterise the LTR-REs of *Stevia rebaudiana* Bertoni. *S. rebaudiana* represents one of the most important crops belonging to the Asteraceae family, native to South America and cultivated today all over the world, as it represents a valid natural alternative to artificial sweeteners. In fact, its leaves produce steviol glycosides (SVglys) with high sweetening power (about 300 times higher than sucrose) and a reduced caloric value. In addition to its sweetening properties, stevia stands out for its numerous pharmacological, antioxidant, anti-inflammatory, and antimicrobial properties [22,23].

Stevia shows wide climatic adaptability, successfully growing in a range of agroecological environments, including semi-humid, subtropical, and temperate zones, which is probably related to a wide genetic variability [24], reflecting its biochemical variability, i.e., regarding the yield of steviol glycosides in the leaves [25]. In its native area, stevia is grown as a perennial plant, especially at high altitudes [26]. In colder regions, it is grown as an annual crop [27].

Stevia breeding aims to produce higher-performing and yielding genotypes [28,29], selecting genotypes capable of adapting to different environmental conditions and characterised by both high levels of secondary metabolites and biomass yield. Therefore, evaluating the genetic variability among different accessions is important for carrying out breeding programmes [30,31]. 

*S. rebaudiana* (2n = 22) has quite a large genome [32]. A chromosome-level assembly of the stevia genome has been released based on Illumina, PacBio, and Hi-C sequencing [33]. The assembly covers 1416 Mb, and more than 80% is made of repeated sequences, especially LTR-REs, which amount to 65.07% of the genome, while other REs and DNA transposons represent only 4.38% and 5.83% of the genome, respectively [33]. However, the LTR-REs of *S. rebaudiana* have only been marginally investigated to date.

LTR-REs can be exploited to discover molecular markers [34,35]. The protocol generally consists of PCR amplification between a conserved retrotransposon feature and other conserved features in the genome. For example, in the case of inter-retrotransposon-amplified polymorphisms (IRAPs) [36], PCR amplification is obtained using a single primer that anneals to LTRs of two adjacent LTR-REs. IRAP markers are dominant, i.e., alleles at a locus are represented by the presence or absence of a DNA fragment, not distinguishing between homozygous and heterozygous loci. Given the ubiquity, abundance, dispersion, and dynamism of LTR-REs, IRAP markers can be conveniently used in species with large-sized genomes, such as *S. rebaudiana*. 

In this work, we identified complete LTR-RE sequences (i.e., from 5′-LTR to 3′-LTR) in the available reference genome of stevia. The retrieved sequences were annotated at the lineage level, and some of them were selected for investigating LTR-RE-related genetic variability of different *S. rebaudiana* accessions by IRAP analysis. 

## 2. Results

### 2.1. Identification and Characterisation of Full-Length LTR Retrotransposons in the S. rebaudiana Genome

**Figure 1:**
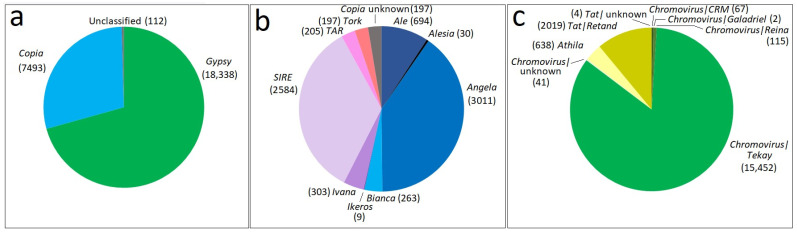
Pie charts of the distribution of full-length LTR-REs in the *S. rebaudiana* genome considering both superfamilies (**a**) and *Copia* (**b**) and *Gypsy* (**c**) lineages. The number of elements for each LTR-RE superfamily or lineage is shown in the brackets.

To identify the LTR-REs, we chose to perform a structural analysis using LTRharvest [37]. This tool appears to be one of the most affordable for LTR-RE isolation [38]. A total of 25,943 full-length LTR-REs (i.e., both LTRs) were isolated from the stevia genome. Overall, 70.68% *Gypsy* and 28.88% *Copia* elements were classified according to protein domain- and homology-based annotation (Figure 1a). The number of *Gypsy* elements was about 2.4-fold higher than the *Copia* elements. However, 0.44% LTR-REs remained undetermined. As an additional highlight, 38.37% of elements showed all protein domains, whereas, in the remaining 61.63% of elements, at least one protein domain was missing; hence, they can be considered transpositionally non-autonomous.

LTR-REs were also annotated at the lineage level. With regard to the *Copia* superfamily (Figure 1b), 40.18% *Angela*, 34.49% *SIRE* and 9.26% *Ale* full-length elements were the most abundant. Additionally, 2.63% of *Copia* elements remained undetermined. The *Angela* lineage was predominant, followed by *SIRE*. Other lineages were less represented. 

As for the *Gypsy* superfamily (Figure 1c), *Chromovirus* elements were the most abundant, with 85.49% of *Gypsy* elements. *Chromovirus* elements are mainly represented by *Tekay* elements (84.26%). Furthermore, 0.22% of *Chromovirus* elements remained undetermined. Regarding the non-*Chromovirus* lineages, 3.48% of *Athila* and 11.01% of *Tat-Retand* elements were characterised. For 0.2% of *Tat* elements, the sublineage remained undetermined. *Tekay* elements were by far predominant in the *Chromovirus* lineage and, more generally, in the *Gypsy* superfamily, followed by *Tat-Retand* elements.

Concerning the genomic abundance of LTR-REs in the stevia genome, mapping Illumina DNA reads to the full-length LTR-REs (see Methods) showed that full-length elements were mapped to 55.8% of the reads used in this analysis, representing 55.8% of the stevia genome. *Gypsy* was the most plentiful superfamily; in fact, *Gypsy* full-length elements accounted for 36.7% and *Copia* elements for 18.7% of the genome. LTR-REs, whose superfamily could not be identified, represented only 0.4% of the genome. The ratio between the genome proportions of *Gypsy* and *Copia* LTR-REs amounts to around 2:1, similar to other Asteraceae, in which *Gypsy* elements are by far the most represented. 

**Figure 2:**
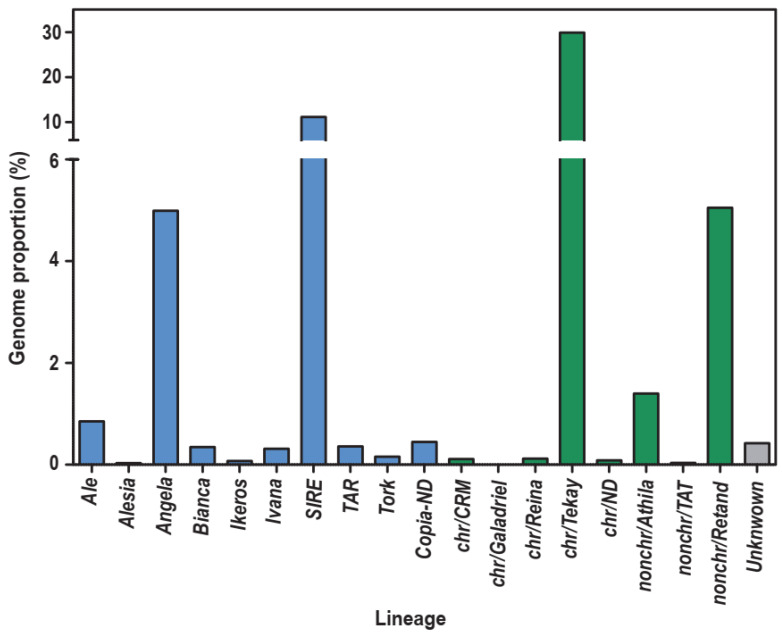
Genome proportions of different LTR-RE lineages in the *S. rebaudiana* genome. Blue: *Copia* lineages; Green: *Gypsy* lineages; Grey: undetermined superfamily; chr: Chromovirus; nonchr: Non-Chromovirus; ND, not determined.

Mapping Illumina DNA reads to the full-length LTR-REs was also performed at the lineage level. Figure 2 reports the genome proportions of each *Copia* and *Gypsy* lineage. The most represented lineages belonging to the *Copia* superfamily were *SIRE* (11.1% of the genome) and *Angela* (5.0%). As for the *Gypsy* superfamily, the most abundant lineage was *Chromovirus/Tekay* (29.9%), followed by *Tat/Retand* (5.0%) and *Athila* (1.4%). All other *Copia* and *Gypsy* lineages accounted for less than 1% of the genome (Figure 2).

### 2.2. Insertion Time Profiles of Stevia LTR Retrotransposons

The proliferation time profiles of the most abundant *Copia* and *Gypsy* LTR-RE lineages in the stevia genome were first inferred by measuring pairwise distances between the LTRs of the same element. In fact, the two LTRs of a retrotransposon should be identical immediately after the insertion event and then undergo mutations over time [39]. Distances were then converted into insertion dates using a mutation rate that was twice the rate calculated for synonymous substitutions in *Helianthus annuus* gene sequences [40,41], presuming that repeats accumulate more mutations than genes as time passes [42]. Although the translation of genetic distances into insertion dates is subject to error, this analysis allowed us to compare proliferation waves among RE lineages.

**Figure 3:**
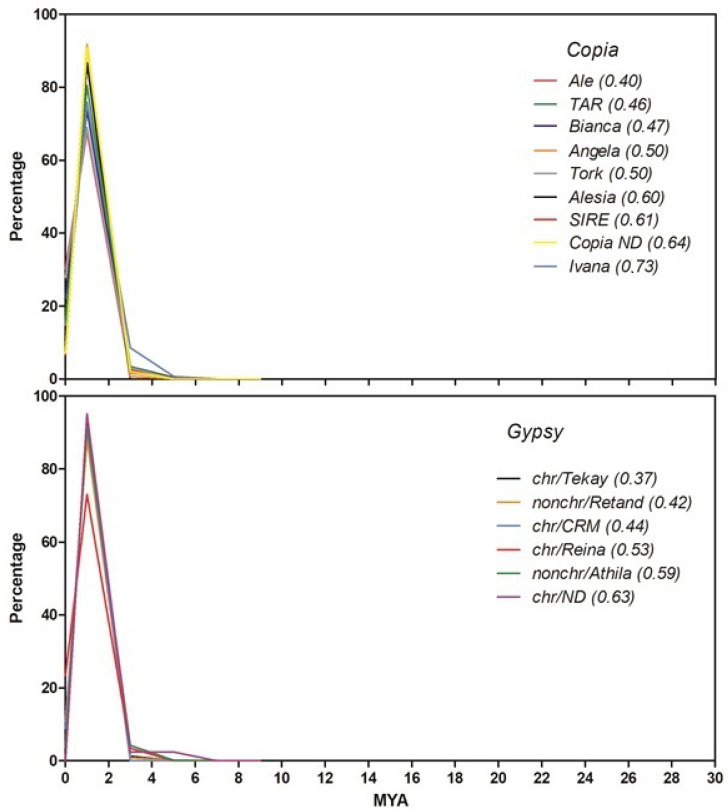
Timing of retrotranspositional activity of eight *Copia* and five *Gypsy* lineages of *S. rebaudiana* based on pairwise comparisons of the LTRs of each full-length element. For each lineage, the average insertion time (in MYA) is reported in parentheses. MYA, million years ago; chr, Chromovirus; nonchr, non-Chromovirus; ND, not determined.

The results of this analysis are reported in Figure 3. All lineages showed a transposition peak at 2 millions of years ago (MYA), although some lineages were younger on average: *CRM* and *Tekay Chromovirus* and *Athila* lineages for the *Gypsy* superfamily and *Ale*, *Angela*, *Ivana* and *Tork* lineages for the *Copia* superfamily (Figure 3).

The isolation of full-length elements is biased towards younger retrotransposons, since ancient elements are subject to more structural changes during evolutionary time. For this reason, insertion time profiles of LTR-RE lineages were also measured using another method, i.e., not based on LTR comparison of full-length elements but by calculating pairwise distances between paralogous RT-encoding sequences belonging to elements of the same lineage [43]. After assembling a sample of Illumina reads, we retrieved RT sequences of six *Copia* or *Gypsy* lineages and used these sequences to isolate DNA sequence reads for comparison. Distances were then translated into insertion dates using the same mutation rate described above. In fact, at each insertion, the new RE copy was identical to its parental element, except for mutations occurring during retrotranscription, which is error-prone [44]; further mutations can accumulate as time passes [42].

**Figure 4:**
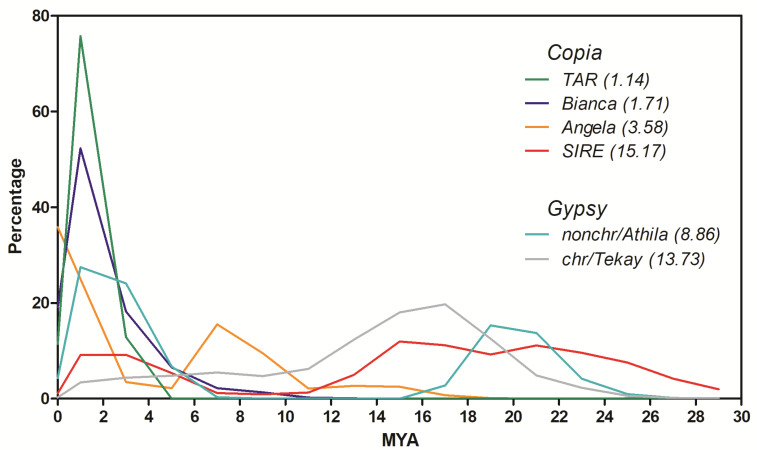
Timing of retrotranspositional activity of four *Copia* and two *Gypsy* lineages of *S. rebaudiana* based on pairwise comparisons of Illumina reads that match RT encoding sequences. The average insertion time (in MYA) for each lineage is reported in parentheses. MYA, million years ago; chr, *Chromovirus*; nonchr, non-*Chromovirus*.

A one million read sample of Illumina reads was clustered using RepeatExplorer (see Methods). Using the same tool, clusters were searched for the presence of RT domains. Subsequently, the reads aligned to the RT domains were pairwise compared, and the proliferation time profiles of four *Copia* and two *Gypsy* lineages (i.e., those of which many aligned reads were available) were calculated. This analysis allowed us to identify different proliferation profiles depending on the different RE lineages analysed (Figure 4).

Two peaks of retrotranspositional activity, one relatively recent (around 1–2 MYA) and one more ancient (from 7–20 MYA, depending on the lineage), were observed. *Copia*/*SIRE* and *Gypsy*/*Athila* lineages showed two proliferation peaks, while *Gypsy*/*Chromovirus*/*Tekay* lineage showed only an ancient peak, and *Copia*/*Bianca* and *Copia*/*TAR* only the most recent peak (Figure 4). Interestingly, this analysis showed that *Copia*/*Angela* elements are the most active in proliferation at present (Figure 4).

### 2.3. IRAP-Based Analysis of Genetic Variability among Stevia rebaudiana Accessions

In another set of experiments, we analysed the genetic variability in a small germplasm collection (25 accessions) of *S. rebaudiana* conserved at our department, performing the IRAP protocol [36] using primers designed on the sequences of LTR-REs. The IRAP protocol detects genomic loci bounded by long terminal repeats of two retrotransposons lying close enough to be amplified by PCR. IRAP PCR fragments can be produced using a single primer when two elements sharing the same LTR sequence are oriented head-to-tail or when two primers are designed on the LTRs of two different head-to-head oriented REs. 

For this analysis, we selected a large group of LTR-REs showing large regions of similarity among at least 511 of the 3011 full-length elements belonging to the *Angela* lineage. Eleven oligonucleotides were designed on the LTRs of these full-length elements. They were tested by PCR on the DNA of three accessions (Supplementary Figure S1). Of the tested oligonucleotides, one (named ANG5+) produced a pattern consisting of several easily scorable bands, with many polymorphic amplification products among the genotypes used in this preliminary experiment, confirming that it identified the LTR region of a highly redundant element. BLAST analysis of the occurrence of this primer in the genome sequence of cv. ‘Zhongshan No. 7’ [33] confirmed the redundancy of the primer, evidencing 2271 copies in the genome. Consequently, primer 5+ was used to analyse all genotypes.

**Figure 5:**
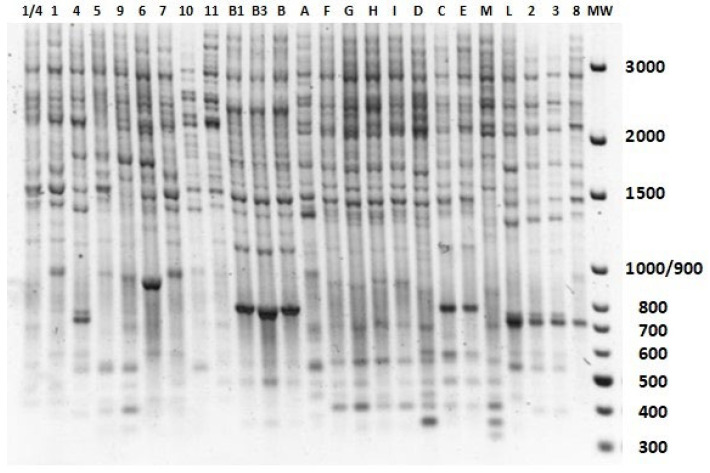
IRAP fingerprints obtained with a primer (ANG5+) targeting LTR-REs belonging to the *Copia*/*Angela* lineage in 25 accessions of *S. rebaudiana*. Genotype codes are listed in Table 3 (see Methods). A molecular weight marker (MW, 100 bp DNA Ladder (Solis Biodyne)) was loaded. The fragment size (bp) is indicated to the right.

Polymorphic bands ranging from 100 to 3000 bp were produced. The electrophoretic patterns using primer 5+ are reported in Figure 5. Nearly identical patterns were obtained in three independent experiments. In rare cases of non-reproducible bands, they were excluded from subsequent analyses. Altogether, primer ANG5+ produced many amplified fragments, apparently related to the redundancy of the element identified by the primer; the observed polymorphism evidence variability in the loci in which these retrotransposons were inserted.

Among the 25 accessions, IRAP fingerprints produced 39 polymorphic bands. Accessions B, B1, and B3 showed an identical electrophoretic pattern, as expected since they are vegetatively propagated clones derived from the same Brazilian accession. In other cases, different accessions showed the same pattern, allowing clarification of their origins. For example, accessions C and E, despite coming from different Italian locations (Ragusa and Nocera), were found to have the same genotype. The same applies to samples 2 and 3 from Brazil (Figure 5). An IRAP fingerprint-based Principal Component Analysis (PCA), illustrating similarities among stevia accessions is reported in Supplementary Figure S2.

### 2.4. Analysis of Population Structure

The numerous bands obtained using primer ANG5+ represented a large number of loci in the stevia genome. In this sense, we used primer ANG5+ related polymorphism to analyse the genome structure of our stevia collection and the occurrence of admixed genotypes.

**Figure 6:**
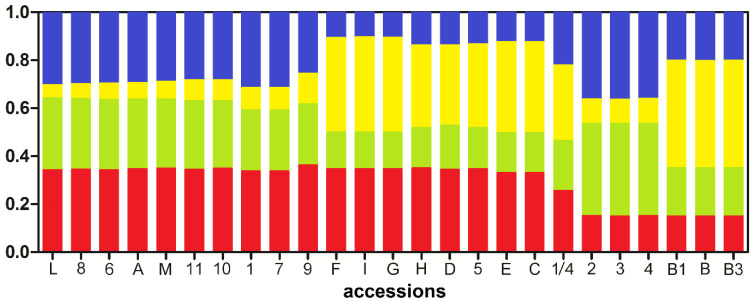
Proportions of the ancestry of 25 *S. rebaudiana* accessions based on K = 4 (where K is the number of initial subpopulations, each indicated with a different colour). Genotype codes are listed in Table 3 (see Methods).

In Supplementary Figure S3, a schematic representation of the IRAP matrix is reported. The analysis of the population structure and the classification of stevia accessions into groups were performed using the Bayesian method in the STRUCTURE software [45]. The number of initial subpopulations (K) was defined as 1 to 25, performing seven replications per run. The maximum value of ΔK was obtained at K = 4 (Supplementary Figure S3). Therefore, in relation to the polymorphisms of full-length *Angela* LTR-REs, the analysed stevia accessions may consist of four ancestral subpopulations (Figure 6). A genotype can be unequivocally assigned to a subpopulation when its admixture coefficient (Qi) is >0.8 for that subpopulation [46,47]. Genotypes with intermediate admixture coefficients (i.e., with Qi < 0.8) are considered admixed. After STRUCTURE was applied, all analysed accessions were classified as admixed (Figure 6).

In order to obtain indications on the possibility of using the IRAP protocol for identifying chromosomal loci related to steviol glycoside content, the stevia genome sequence was investigated to establish whether genes potentially involved in steviol glycoside metabolism lie close to the loci individuated by the IRAP primer (ANG5+), i.e., within 100,000 bp upstream and 100,000 downstream. In particular, we collected a repertoire of 103 genes involved in steviol glycoside metabolism, as identified by BLAST analysis on the SwissProt manually curated a database against stevia protein sequences [33] (Simoni, personal communication) and searched for the occurrence of the ANG5+ primer sequence within 100,000 bp upstream and downstream of each gene. The occurrence of the ANG5+ primer was recorded in proximity to 35 of 103 SVglys genes, whereas a total of 16 ANG5+ primer was detected in proximity to 103 randomly collected genes. The Chi-square test showed a significant difference (*p*-value = 0.0036) in the occurrence of ANG5+ primer in the frame of 100,000 bp surrounding SVglys genes. Table 1 reports the gene families to which these 35 genes belong. This result suggests that the described IRAP protocol could be potentially used for identifying steviol glycoside content-related loci in the stevia genome. We are collecting a large number of stevia genotypes to carry on this analysis.

## 3. Discussion

### 3.1. Repetitive Component of the S. rebaudiana Genome

DNA sequencing of long sequence reads and new sequence-assembling strategies permit the achievement of much more precise genome sequences than before. In particular, genomes sequenced using long sequence reads allow for more precise and reliable structural identification and characterisation of repeated elements [38,48]. 

Our analyses allowed us to identify and characterise the full-length LTR-REs in the *S. rebaudiana* genome. The identification of repetitive DNA has already been reported by Xu et al. [33]. Concerning LTR-REs, Xu et al. [33] reported the identification of 653,092 elements, but they did not specify whether this number also included LTR-RE fragments. In fact, during genome evolution, LTR-REs are subject to rearrangements, producing several retrotransposon remnants [49,50]. The large difference between the number of elements reported by Xu et al. [33] and the number of full-length LTR-REs identified in our experiments (25,943) suggests that many of the elements were identified by Xu et al. [33] are RE fragments/remnants. As a matter of fact, in sunflower, where LTR-REs show an abundance similar to that observed in the stevia genome, the number of full-length elements is of the same order of magnitude as that found in stevia [51].

Full-length elements were annotated at the superfamily and lineage levels. Similar to other Asteraceae, such as the sunflower [52], full-length elements of the *Copia* superfamily are much less frequent than *Gypsy*. All main lineages of the LTR-REs are present in the stevia genome. Concerning the *Copia* superfamily, *Angela* elements were the most frequent, followed by *SIRE* LTR-REs, as observed in other Asteraceae, such as *Lactuca sativa* [51]. For the *Gypsy* superfamily, the most frequent lineage was by far *Chromovirus*, sublineage *Tekay*, as already reported for another Asteraceae genus, *Hieracium* [53]. All these species have medium-large genome sizes [51,53]. The prevalence of one superfamily or of one lineage over the others is not related to the genome size of the species [54], as expected, since REs are autonomous in replication.

The genome abundance and putative insertion age were estimated for each full-length LTR-RE. Overall, full-length LTR-REs amount to 55.8% of the genome, as shown by mapping Illumina reads to their sequences. By masking the genome with a collection of repeat sequences, Xu et al. [33] estimated the abundance of LTR-REs to be 69.4% of the stevia genome. Presumably, the difference in LTR-RE abundance estimation is related to the occurrence of many RE remnants and fragments (i.e., incomplete elements) belonging to old families with degenerated sequences that are not recognised by the software that predicts only full-length LTR-REs, as observed in other species [55].

Mapping analysis showed that the abundance of sequences related to *Gypsy* full-length REs is around two-fold that of *Copia* ones, confirming the data reported by Xu et al. [33]. The ratio between the abundance of the *Gypsy* and *Copia* sequences is highly variable and differs between species [54]. In Asteraceae, the family to which the genus *Stevia* belongs, this ratio is generally higher than 1, for example, in sunflower [56,57] or Hieracium [53], although there are also genera, such as *Melampodium* [58], *Anacyclus,* and *Heliocauta* [59], in which there are more *Copia* than *Gypsy* elements.

Concerning the *Gypsy* lineages, the most frequent lineages in the collection of full-length LTR-REs, *Chromovirus/Tekay*, non-*chromovirus/Retand* and non-*chromovirus/Athila* were also the most abundant in the genome. In contrast, concerning the *Copia* superfamily, although *Angela* full-length elements are the most numerous, the most abundant lineage is by far *SIRE*, covering around 10% of the genome, while the *Angela* lineage accounts for around 5%. This indicates the occurrence, in the stevia genome, of many *SIRE* remnants and fragments. In fact, *SIRE* LTR-REs are apparently the most ancient, as shown by the insertion time profiles calculated comparing RT sequences. Presumably, many ancient *SIRE* REs have undergone many mutation events, remaining in the genome as fragmented elements (i.e., not full-length) not recognised by the tools based on *de novo* detection. It should be noted that *SIRE* elements are the most abundant in many Asteraceae genera, such as *Cynara*, *Artemisia*, *Carthamus,* and *Chrysanthemum* [51].

Further characterisation of stevia LTR-REs consisted of estimating the insertion time profiles of different RE lineages. Insertion time calculation based on LTR sequence comparison [39] showed that the insertions of all the isolated full-length elements are relatively recent, with a transposition peak at 1–2 MYA. It is, however, presumable that insertion time calculated on LTR comparison of full-length elements is biased because the more ancient the element, the more it does not maintain structural integrity and hence cannot be identified by tools analysing structural features.

For this reason, another analysis was performed on a sample of Illumina sequence reads complementary to retrotranscriptase sequences, according to Piegu et al. [43]. The insertion time profiles of those lineages for which a reliable number of sequences were available showed how the proliferation of REs in *S. rebaudiana* occurred mainly in two distinct evolutionary periods, with two peaks, one at 5 MYA and the other more ancient, from 14 to 24 MYA, depending on the lineage. In the case of *Chromovirus/Tekay* elements, which are by far the most abundant in the genome, their abundance seemed to be especially related to the oldest proliferation peak. The *Angela* lineage does not seem to have reached the peak of proliferation yet.

### 3.2. Use of LTR-Retrotransposons for Genetic Variability Analyses

Due to their repetitive and dispersed nature, REs are very suitable sequences to exploit for the analysis of genetic variability [35]. An IRAP protocol [36] was developed to evaluate the genetic variability of 25 *S. rebaudiana* accessions relative to a specific LTR-RE. This RE belongs to the *Angela* lineage of the *Copia* superfamily. It was selected because it is abundant in the genome and belongs to a lineage that appears to still be active. PCR analyses using a primer designed on this element produced electrophoretic patterns characterised by many bands of different lengths, many of which were polymorphic.

This analysis allowed the identification of cases of identity between some accessions, for example, the pair formed by accessions 1 (MA pt1) and 7 (MA pt3), and that formed by accessions 2 (MA 13/1) and 3 (MA pt2), all of Brazilian origin. For other accessions, PCA made it possible to establish relationships of strict similarity. For example, the genotypes F (CO), I (BR1) and G (BR5) are very close, despite F being of Israeli origin and I and G being Brazilian accessions. Moreover, Brazilian genotypes 5 (MA pt5) and 9 (SV1) resulted similar to Israeli genotype D (SL), sharing many of their polymorphic bands, suggesting a probable common origin, which requires further investigation. 

After performing a population structure analysis and considering the genomic regions identified by the polymorphisms of the *Copia*/*Angela* LTR-RE, we hypothesised the existence of four ancestral subpopulations from which our 25 accessions originated. All accessions, which come from different regions of the world where *S. rebaudiana* is cultivated (Brasil, Paraguay, Israel, and Italy), were found to be admixed, with no relation to geographic origin, indicating that the 4 original subpopulations probably had the same geographic origin.

Many more copies of the sequence identified by the primer used for obtaining IRAP fingerprints were found in the stevia genome in the proximity of genes involved in steviol glycoside metabolism, for example, encoding many UDP-glycosyltransferases, than in the proximity of randomly selected genes. It is known that the presence of non-coding sequences, such as retrotransposons, may affect the expression of proximal genes. In fact, an inserted retrotransposon may epigenetically change the expression [60] or act as a distal enhancer of genes along the chromosomal locus, even at distances of 1000 base pairs. For example, the maize teosinte-branched1 gene expression pattern differed depending on the presence of a hopscotch retrotransposon at more than 60,000 bp from the tb1 gene [61]. Our analysis suggests the possibility of using the IRAP protocol described in this article to identify chromosomal loci involved in steviol glycoside content.

## 4. Materials and Methods

### 4.1. Full-Length LTR-Retrotransposons Collection and Characterisation

The stevia genome assembly (https://doi.org/10.6084/m9.figshare.14169491.v1; accessed on 5 July 2021) [33] was scanned for Class I full-length LTR-REs. The elements were identified using EDTA v1.9.3 [62]. EDTA implemented a combination of LTR_FINDER v1.06 [63], LTRharvest v1.5.10 [48], and LTR_retriever v2.5 [64]. All implemented programme parameters were automatically set, as reported in the default pipeline [62].

The identified full-length LTR-REs were submitted to domain-based annotation using DANTE v1.0.0, available on the RepeatExplorer2 Galaxy-based website (https://repeatexplorer-elixir.cerit-sc.cz/galaxy/; accessed on 8 November 2021). The annotation process was performed with default parameters using the REXdb of transposable element protein domains [19] and a BLOSUM80 scoring matrix. The protein matches were filtered by significance using the parameters provided by the platform. 

To reduce the number of uncharacterised full-length LTR-REs, we performed blastn and tblastx processes [65] between uncharacterised and characterised elements. BLAST processes were performed with default parameters.

The insertion profile time course of the different LTR-RE lineages was studied by calculating the distributions of pairwise divergence comparisons of the 5′- and 3′-LTRs. LTR pairwise alignments were calculated using a stretcher of the EMBOSS v6.6.0.0 suite, applying the Kimura two-parameter model of sequence evolution [66]. Distance matrices were prepared using distmat tools of the same suite [67]. The insertion times of each LTR-RE were estimated using a mutation rate of 2 × 10^−8^, which is two-fold the rate calculated for synonymous substitutions in gene sequences in *Helianthus annuus* [42] because LTR-REs accumulate more mutations with time compared to gene sequences. Peaks in frequency distribution were interpreted as transposition burst events, with those peaks associated with lower divergence values considered to represent relatively recent proliferation events [17,42].

LTR-RE insertion time profiles were also calculated using another method based on RT-encoding sequences [41,43,68]. First, a database of stevia repeats was produced using the RepeatExplorer on a sample of one million randomly selected paired-end reads. Briefly, Illumina DNA sequences of *S. rebaudiana* were collected from the NCBI Sequence Read Archive (NCBI, WA, USA, https://www.ncbi.nlm.nih.gov/sra; accessed on 5 July 2021). The ID code of the sequence read set is SRR6792730. FastQC v0.11.5 [69] was run to check the sequence reads for quality. Trimmomatic v0.33 [70] was used to remove Illumina adapters and low-quality regions, with the following parameters: ILLUMINACLIP: 2:30:10, SLIDINGWINDOW: 4:28, HEADCROP: 15, and MINLEN: 85. 

Assembled nucleotide sequences encoding the RT domains (at least 150 nt in length) were selected from clusters related to the LTR-REs of the different lineages using the DANTE protein domain search tool of RepeatExplorer2. The time course was studied by calculating the distributions of pairwise divergence values for Illumina reads aligned with the RT domain-encoding sequences for the different lineages. Briefly, Illumina 85-nt reads were aligned to the RT sequences using CLC Genomics Workbench v9.5.3 (CLC-BIO, Aarhus, Denmark) with the following parameters: mismatch cost = 1, insertion/cost = 1, length fraction = 0.9, and similarity fraction = 0.8. Then, pairwise divergence values between reads were calculated using MEGA v10.1.8 [71] under the Kimura two-parameter model of sequence evolution [66]. Kimura distances were converted to MYA using the same substitution rate as above.

### 4.2. Plant Materials and DNA Isolation

A collection of 25 accessions of *S. rebaudiana* was analysed, as reported in Table 2. This collection is available at the Department of Agriculture, Food, and Environment (DAFE), University of Pisa. Genomic DNA was extracted from young leaves (0.5 g fresh weight) of *S. rebaudiana* plants, as described by Doyle and Doyle [72].

### 4.3. IRAP Analysis

OLIGO v7.0 software [73] was used to design primers related to the DNA sequences belonging to the LTRs of a *Copia* RE. The primers are reported in Table 3. Genomic DNA from the 25 stevia accessions was used as templates.

**Table 3:** List of primers designed on putative LTRs of a group of *Copia*/Angela full length LTR-REs and used for IRAP analysis.

Primer Code	Sequence
ANG RNase+ (forward)	5′-ATGGGACTTCGWTATTCTAGTG-3′
ANG 1− (reverse)	5′-TTTGAGAGCGGGTCAGTCCAA-3′
ANG 3− (reverse)	5′-CCATTCAATAACATCATCATCT-3′
ANG 44− (reverse)	5′-TTATTTACTTATGTTATTTACCA-3′
ANG 44+ (forward)	5′-ATTGGTAAATAACATAAGTAAAT-3′
ANG 4− (reverse)	5′-CACAAGCTTGTATACCCCAAG-3′
ANG 5+ (forward)	5′-TTCAAGAATCACACCCTCTA-3′
ANG 55+ (forward)	5′-TCATAACCTAGCCAAGACCT-3′
ANG 55− (reverse)	5′-AGGTCTTGGCTAGGTTATGA-3′
ANG 6+ (forward)	5′-AACAAACGCGACAAACTAAAAC-3′
ANG gag− (reverse)	5′-CAATTCTCAAGTTTCGATACCA-3′

PCR reactions were carried out as described by Vukich et al. [74] in a reaction mixture (volume 20 μL) containing 50 ng genomic DNA, 2.5 mM MgCl2, 0.5 μM primers, and 1.25 U Taq FirePol (Biodyne) DNA polymerase. Thermocycling was performed at 94 °C for 3 min, 30 cycles at 94 °C for 30 s, 55 °C for 30 s, and 72 °C for 120 s, and a final extension at 72 °C for 7 min. PCR products were visualised on Gel-Red (Biotium) stained 2% agarose gel.

Each electrophoresis was repeated three times, and fingerprints were scored to prepare binary matrices. IRAP fingerprints were analysed by comparing the presence of amplification fragments among the analysed genotypes. The value of “1” or “0” was assigned to each amplification fragment (visualised as a single band on the IRAP fingerprint) in case of presence or absence, respectively, in the analysed accession. Each band was presumed to be representative of a single locus [75]. Non-reproducible bands were rare and excluded from the analyses, along with weak bands.

In order to visualise genetic distance and similarities among genotypes, a principal component analysis (PCA) was performed on the IRAP matrix by using Graphpad Prism (v9.0.0) (GraphPad Software, Inc., La Jolla, CA, USA).

### 4.4. Analysis of Population Structure

To detect mixed genotypes, the population structure was analysed using the Bayesian method with the STRUCTURE v2.3.4 software package [45], based on the IRAP matrix. The number of initial subpopulations (K) was defined from 1 to 25, and five replications were performed per run. The length of the burn-in period was set to 50,000, and the number of Markov Chain Monte Carlo replications was set to 100,000. An admixture model and correlated allele frequencies were chosen. The results were run in STRUCTURE Harvester [76] to choose the most likely number of initial subpopulations, applying the delta K (ΔK) method based on calculating the logarithm of the likelihood for each K (Ln P (D) = L (K)) [77] and the ΔK statistic, the latter of which is based on the secondary rate of change in likelihood (ΔK = (L″(K))/standard deviation) [78]. Using this method, the probability of a slope breaks at the point where the number of hypothetical initial subpopulations is at the maximum point of likelihood.

### 4.5. Analysis of Proximity between Retrotransposons and Genes Involved in Steviol Glycoside Accumulation

A repertoire of 103 genes involved in steviol glycoside accumulation [33] was localised in the *S. rebaudiana* genome [33] through a BLAST analysis of stevia protein sequences against the SwissProt database. After that, another blastn analysis was performed against the *S. rebaudiana* genome using the primer designed on the LTR-RE for the IRAP analysis to evaluate their overall occurrences. The blastn process was performed using the -task “blastn-short” option for short sequences. Then, 100,000 bp upstream and downstream of each of the 103 genes involved in steviol glycoside accumulation were collected and intersected with the blastn results of the LTR-RE primer using BEDTools v2.30.0 [79]. Another 103 genes were randomly selected in the stevia genome by using an in-house python script and the occurrence of the IRAP primer in their proximity (100,000 bp upstream and downstream of each of the 103 genes) was analysed as above. The difference in the occurrence of primers in the frame of 100,000 bp surrounding 103 SVglys genes and 103 randomly collected genes was performed by using the Chi-square test.

## 5. Conclusions

This work reports on the identification and characterisation of the LTR-REs of *Stevia rebaudiana*, a cultivated species of great interest for its application in the sweetener industry. We also verified the possibility of producing and using molecular markers related to repeated sequences in this species. Overall, these data may be useful for the annotation of genomic sequences and for the evaluation of genetic variability to guide stevia breeding.

## Figures and Tables

**Table 1:** Number of genes belonging to gene families involved in steviol glycoside metabolism found in proximity (within 100,000 bp upstream and downstream of each gene) of sequences complementary to ANG5+ IRAP primer.

Gene Family	Number of Genes
UDP-glycosyltransferase	18
Ent-kaurene oxidase	5
Geranylgeranyl pyrophosphate synthase	3
Methyl-erythritol-phosphate cytidylyltransferase	3
Hydroxy-methylbutenyl diphosphate synthase (ferredoxin)	2
Deoxy-xylulose-phosphate reductoisomerase	1
Deoxy-xylulose-phosphate synthase	1
Isopentenyl-diphosphate Delta-isomerase	1
Methyl-erythritol cyclodiphosphate synthase	1

**Table 2:** ID code, name, and origin of 25 *S. rebaudiana* accessions used in this research.

ID Code	Accession Name	Origin	Cultivated in
1/4	MA1/4	Brasil	pot
1	MApt1	Brasil	pot
2	MA13/1	Brasil	pot
3	MApt2	Brasil	pot
4	MA10/1	Brasil	pot
5	MApt5	Brasil	pot
6	MA7/3	Brasil	pot
7	MApt3	Brasil	pot
8	MApt4	Brasil	pot
9	SV1	Brasil	pot
10	Criolla	Paraguay	pot
11	Sweet Herb	Paraguay	pot
A	PL	Israel	field
B	BR16	Brasil	pot
C	RGm	Italy	field
D	SL	Israel	field
E	Num	Italy	pot
F	CO	Israel	field
G	BR5	Brasil	field
H	SW30	Italy	field
I	BR1	Brasil	field
L	MASV4-2/2	Brasil	pot
M	Eirete	Paraguay	pot
B1	BR16	Brasil	pot
B3	BR16	Brasil	pot

## Data Availability

Not applicable.

## Supplementary Materials

The following are available online at https://www.mdpi.com/article/10.3390/ijms23116220/s1.

## Abbreviations

LTR	Long-terminal repeats
REs	Retrotransposons
TEs	Transposable elements
MYA	Million Years Ago
SVglys	Steviol glycosides
