# Supplementary material for: Characterisation of LTR-Retrotransposons of Stevia rebaudiana and Their Use for the Analysis of Genetic Variability
Source: Int J Mol Sci. 2022 Jun 1;23(11):6220. doi: 10.3390/ijms23116220 (PMC9181549; doi:10.3390/ijms23116220)
Supplement: Supplementary file 1 [file ijms-23-06220-s001.zip › ijms-1672807-supplementary/ijms-1688985_figure1.pdf]

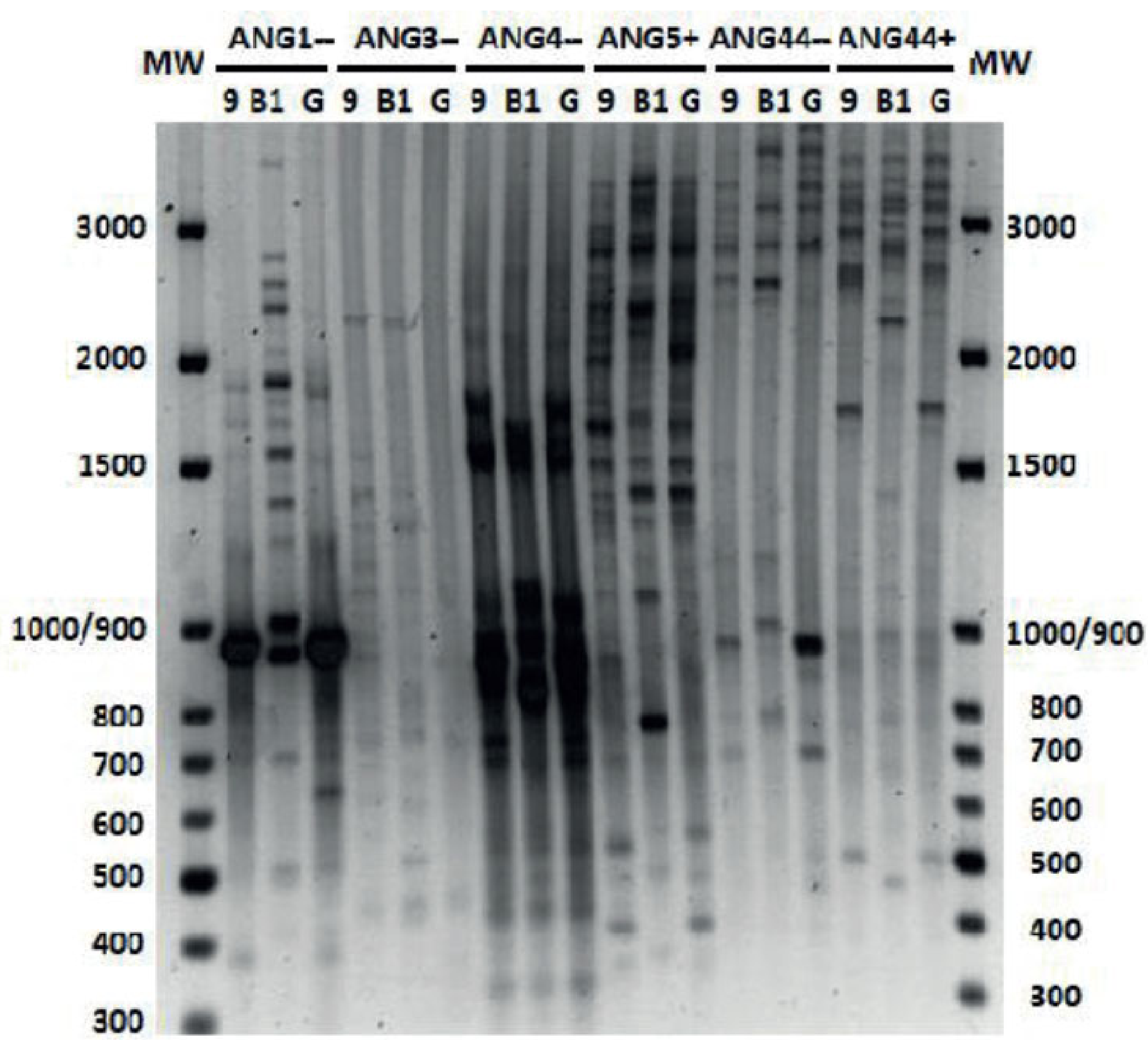

Figure S1. IRAP fingerprints obtained with 6 primers (ANG1– to ANG44+) targeting LTR-REs belonging to the *Copia/Angela* lineage in 3 accessions of *S. rebaudiana.* Accession codes are listed in Table 2. A molecular weight marker (MW, 100 bp DNA Ladder (Solis Biodyne)) was loaded.
