# Supplementary material for: Characterisation of LTR-Retrotransposons of Stevia rebaudiana and Their Use for the Analysis of Genetic Variability
Source: Int J Mol Sci. 2022 Jun 1;23(11):6220. doi: 10.3390/ijms23116220 (PMC9181549; doi:10.3390/ijms23116220)
Supplement: Supplementary file 1 [file ijms-23-06220-s001.zip › ijms-1672807-supplementary/ijms-1688985_figure2.pdf]

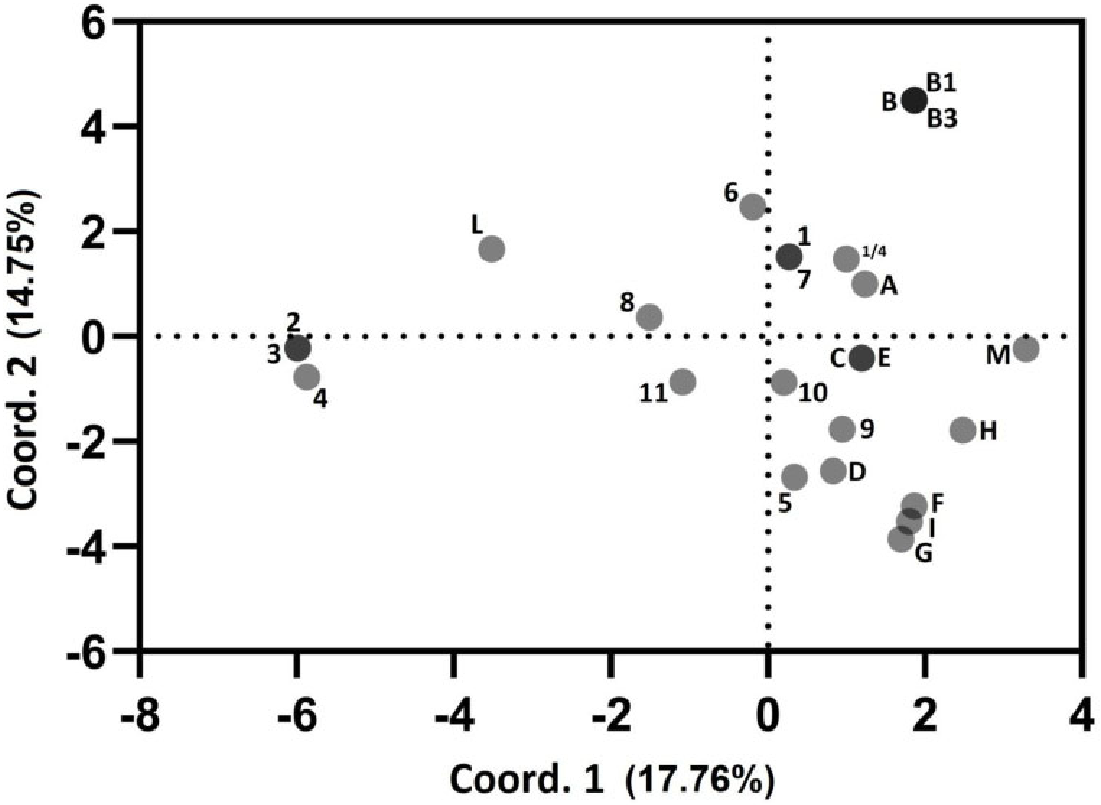

Figure S2. PCA plot based on IRAP matrix of 25 accessions of *Stevia rebaudiana*. Accession codes according to Table 3. Grey dots indicate single accessions, black dots indicate an identical position of more than one accession.
