# Supplementary material for: Characterisation of LTR-Retrotransposons of Stevia rebaudiana and Their Use for the Analysis of Genetic Variability
Source: Int J Mol Sci. 2022 Jun 1;23(11):6220. doi: 10.3390/ijms23116220 (PMC9181549; doi:10.3390/ijms23116220)
Supplement: Supplementary file 1 [file ijms-23-06220-s001.zip › ijms-1672807-supplementary/ijms-1688985_figure3.pdf]

Genotype:	
1/4	0 1 1 1 0 1 0 1 1 0 0 0 1 0 0 0 1 1 1 0 0 0 0 0 0 0 0 0 0 0 1 0 1 1 0 0 0 0 0
1	1 1 1 1 1 1 0 1 1 0 0 0 1 0 0 1 1 1 1 0 1 0 0 0 0 0 0 0 0 1 0 0 1 1 1 0 0 0 0
6	0 0 0 0 0 1 0 0 1 0 0 1 1 0 0 1 0 1 0 1 0 0 1 0 0 0 0 0 0 1 0 0 0 1 1 0 0 0 0
7	1 1 1 1 1 1 0 1 1 0 0 0 1 0 0 1 1 1 1 0 1 0 0 0 0 0 0 0 0 1 0 0 1 1 1 0 0 0 0
B	0 0 0 1 0 1 0 1 1 0 0 0 1 0 0 1 1 1 0 0 0 0 0 0 1 0 0 0 0 0 1 0 1 0 0 1 0 0 0
B1	0 0 0 1 0 1 0 1 1 0 0 0 1 0 0 1 1 1 0 0 0 0 0 0 1 0 0 0 0 0 1 0 1 0 0 1 0 0 0
B3	0 0 0 1 0 1 0 1 1 0 0 0 1 0 0 1 1 1 0 0 0 0 0 0 1 0 0 0 0 0 1 0 1 0 0 1 0 0 0
C	1 1 1 1 1 1 0 1 0 0 0 1 1 0 0 0 1 1 0 0 0 0 0 0 1 0 0 1 1 1 1 0 1 0 1 0 0 0 0
E	1 1 1 1 1 1 0 1 0 0 0 1 1 0 0 0 1 1 0 0 0 0 0 0 1 0 0 1 1 1 1 0 1 0 1 0 0 0 0
8	1 0 0 0 1 1 0 0 0 1 0 0 1 0 1 0 1 1 1 0 1 0 0 1 0 0 1 0 1 0 0 1 0 0 0 0 0 0 0
F	0 0 1 0 1 0 0 1 0 0 0 1 1 0 1 0 1 1 1 0 0 1 0 0 0 0 0 1 0 0 1 0 1 1 1 0 0 1 0
D	0 1 1 0 1 0 0 1 0 0 0 1 1 0 1 0 1 1 0 1 0 0 0 1 0 0 0 1 0 1 1 0 1 1 1 0 1 0 0
G	0 1 1 0 1 1 1 1 0 0 0 1 1 0 1 0 1 1 1 0 0 1 0 0 0 0 0 1 1 0 1 0 1 1 1 0 0 1 0
M	0 0 0 1 0 0 0 1 0 0 1 0 1 0 1 1 0 1 1 1 0 0 0 1 0 0 0 1 0 0 1 0 1 1 1 0 1 0 1
H	0 0 0 1 0 1 0 1 0 0 0 1 1 0 1 0 1 1 0 0 1 0 0 1 0 0 0 1 1 0 1 0 0 1 1 0 0 1 0
I	0 0 1 1 1 1 1 1 0 0 0 1 1 0 1 0 1 1 0 0 0 1 0 0 0 0 0 1 1 0 1 1 1 1 1 0 0 1 0
10	1 1 0 1 0 1 1 1 1 0 1 1 0 1 0 0 1 1 0 0 1 0 0 0 0 0 0 1 0 0 1 1 0 1 1 0 0 1 0
11	1 1 1 1 1 0 1 1 0 1 0 0 1 0 0 0 1 1 0 1 1 0 0 1 0 1 0 1 0 1 1 0 1 1 0 0 0 0 0
A	1 0 0 1 0 1 0 0 0 0 0 1 0 1 0 0 0 1 1 0 1 0 0 0 0 0 0 1 1 0 1 1 1 1 0 1 0 0 0
5	1 1 1 1 0 0 1 1 0 0 0 1 1 0 1 0 1 1 0 0 1 0 0 0 0 0 0 0 1 0 1 1 0 1 1 0 0 1 0
2	0 1 1 0 1 0 1 0 1 0 0 0 1 1 0 0 1 0 0 0 0 0 0 0 0 1 1 0 0 1 1 1 0 0 1 0 0 0 0
3	0 1 1 0 1 0 1 0 1 0 0 0 1 1 0 0 1 0 0 0 0 0 0 0 0 1 1 0 0 1 1 1 0 0 1 0 0 0 0
L	0 0 1 1 0 1 1 0 0 1 0 0 0 1 0 1 0 1 1 1 1 0 0 1 0 1 1 0 0 1 0 1 0 1 0 0 0 0 0
4	0 1 1 0 1 0 1 0 0 0 0 0 1 1 0 0 1 0 0 0 0 0 0 0 0 1 1 0 0 1 1 1 0 0 1 0 0 0 0
9	0 0 1 1 0 1 0 0 0 0 0 1 1 0 0 0 0 1 1 1 1 0 0 1 0 1 0 1 1 0 1 1 1 1 1 0 0 1 0

Figure S3. A Binary matrix of IRAP bands used for STRUCTURE analysis. The scheme is derived from electrophoreses of PCR fragments produced using the primer ANG5+ in 25 stevia accessions.

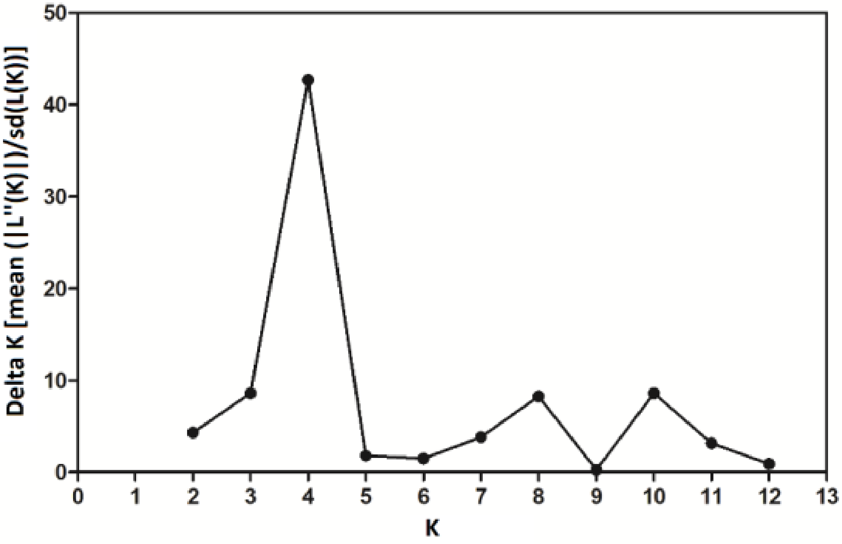

Figure S3. B Bilateral chart to access the optimal number of sub-population in studied stevia accessions (K = 4), based on the above reported 39 polimorphic loci.
